# Cancer Stem Cell Marker CD147 Expression in Erosive Oral Lichen Planus Compared to Moderately and Severely Dysplastic Leukoplakia

**DOI:** 10.3390/reports7030077

**Published:** 2024-09-15

**Authors:** Vasileios Zisis, Nikolaos Nikitas Giannakopoulos, Athanasios Poulopoulos, Marc Schmitter, Dimitrios Andreadis

**Affiliations:** 1 Department of Prosthodontics, University of Wuerzburg, 97070 Würzburg, Germany; 2 Department of Oral Pathology and Medicine, Aristotle University of Thessaloniki, 54124 Thessaloniki, Greece

**Keywords:** stem cells, cancer, lichen planus, oral, CD147

## Abstract

Oral lichen planus is a frequent, chronic autoimmune disease that affects the oral mucosa and is characterized as an oral potentially malignant disorder. The aim of our study is to examine the presence of CSCs bearing CD147 (a marker related to local inflammation and associated with various cancers) through immunohistochemistry in oral lichen planus (OLP) compared to oral leukoplakia (OL) and healthy tissues. These findings could contribute to clinical practice by providing a marker for the prognostic assessment of OLP lesions with regards to their potentially malignant nature. The study sample consisted of paraffin-embedded oral mucosa specimens from the archives of the Department of Oral Medicine/Pathology, School of Dentistry, Aristotle University of Thessaloniki, Greece during the period 2009–2019. The study sample contained 24 cases of OLP (14 erosive and 10 reticular) and 30 cases of oral leukoplakia, which were compared to 5 normal oral epithelium samples derived from healthy epithelium adjacent to fibromas from other cases. Cell membrane staining of CD147 was observed mostly in the basal and parabasal cell layer. The statistically significantly higher expression of CD147 in the erosive lichen planus subgroup than in the moderately and severely dysplastic leukoplakia subgroup (*p* = 0.01) constituted the most important finding of this study. The characteristic expression of CD147 in erosive OLP suggests the presence of epithelial cells with CSC characteristics, but its lower expression in oral leukoplakias suggests a more intense relation of the CD147 marker with inflammation rather than with oral dysplastic progression.

## 1. Introduction

Current terminology refers to oral precancerous lesions as “oral potentially malignant disorders (OPMDs)”. OPMDs include oral mucosal lesions, which exhibit an increased risk for malignant transformation compared to healthy mucosa [1]. Oral leukoplakia (OL) constitutes the most common OPMD [2], whereas oral lichen planus (OLP) and oral lichenoid reaction (OLR) are considered to be relatively rare OPMDs [3]. 

Lichen planus (LP) is a frequent, chronic mucocutaneous autoimmune disease that often affects the oral mucosa but can also affect the skin, scalp, genitalia and nails [4,5]. The cutaneous lesions expand like lichens (algae and fungi) on rocks [6]. Of course, despite its name, lichen planus is not a fungal condition but an immunologically mediated disorder. Oral lichen planus is a progressive, inflammatory condition that may affect the oral mucosa in its entirety.

Oral lichen planus (OLP) more commonly affects middle-aged patients than children [7,8], especially females during the sixth and seventh decade of their lives [9]. The prevalence of OLP, depending on the population, ranges from 0.02% to 1.2% [5]. Habits play a major role regarding the relative risk: 0.3% in nonsmokers, 3.7% in people with mixed oral habits, 13.7% in smokers and people who chew tobacco [10]. All racial groups are affected [10]. OLP is more frequent than the cutaneous form, persists longer and responds less successfully to therapeutic measures [11]. Malignant transformation rates range from 0.2% up to 12.5%, being up to 60 times higher than rates in the general population [12,13,14]. On average, it ranges from 0% to 5.3% [15,16]. Furthermore, in cases of oral squamous cell carcinomas (OSCCs) associated with OLP, the risk of lymph node metastases and multiple primary metachronous tumors of the oral cavity is increased [17,18].

OLP incidence is higher in medically compromised patients. The medical history of OLP patients may include autoimmune disorders, such as ulcerative colitis, myasthenia gravis, primary biliary cirrhosis, chronic active hepatitis, thymoma [19], celiac disease, Crohn’s disease [20], psoriasis and lichen sclerosis [21] or neoplasms, such as breast cancer and metastatic adenocarcinoma [21], indicating either an underlying intra-correlation or simply an underlying comorbidity among these disorders. Special attention is required for the correlation between diabetes mellitus and hypertension, on one hand, and OLP, on the other hand [22,23,24]. This established association can also be considered a syndrome, the so-called Grinspan’s syndrome [25]. Also, OLP combined with vulval LP and vaginal LP (anal LP is also a possibility) constitute a clinical triad known as vulvo-vagino-gingival syndrome, a form of erosive plurimucosal LP [26]. The correlation between OLP and hepatitis is ambivalent; the current data support an association with hepatitis C but not hepatitis B, thus proposing the screening of OLP patients for hepatitis C [27]. The overall prevalence of hepatitis C in OLP patients varies from 0% to over 60% [11]. OLP is also associated with the viruses HIV, HHV-6, HPV and EBV [28,29,30,31,32].

It has been suggested that the progression of OPMD to cancer is partly influenced by the presence of cancer stem cells (CSCs). Cancer stem cells exhibit cancer stem markers that control the molecular processes responsible for maintaining the stem cell phenotype. CD147 (cluster of differentiation 147) is classified as a stemness marker, which is crucial for preserving the status of stem cells. CD147, also known as extracellular matrix metalloproteinase inducer (EMMPRIN), is a glycosylated protein that falls under the immunoglobulin class. CD147 exists in two distinct forms: the transmembrane form and the soluble form [33]. The transmembrane region comprises two segments: an extracellular domain and a cytoplasmic tail. This region plays a crucial role in the initiation or activation of MMP. On the other hand, the soluble region has been demonstrated to be a valuable indicator for hepatocellular cancer. A correlation has been discovered between elevated CD147 expression and an unfavorable prognosis in patients with OSCCs [34]. This study aims to examine the expression pattern of CD147 in oral lichen planus (OLP) in comparison to oral leukoplakia (OL) and normal epithelium. These findings could contribute to clinical practice by providing a marker for the prognostic assessment of OLP lesions with regards to their potentially malignant nature.

## 2. Materials and Methods

### 2.1. Tissues

Paraffin-embedded samples of 24 cases in the oral lichen planus group, 30 cases in the oral leukoplakia group and 5 cases in the normal oral epithelium group (used as a control) were used. The samples were retrieved from the archives of the Department of Oral Medicine/Pathology, School of Dentistry, Aristotle University of Thessaloniki, Greece during the period 2009–2019. The inclusion criterion was the presence of an adequate quantity of paraffin-embedded tissue, whereas the exclusion criterion was the opposite.

The study was conducted in accordance with the guidelines of the Research and Ethics Committee of the Aristotle University, School of Dentistry and the Declaration of Helsinki II. The present study was approved by the Ethics Committee of the School of Dentistry, Aristotle University of Thessaloniki, Greece at its meeting on 3 July 2019 with the protocol number 8/03.07.2019. 

The tissue samples were divided into the lichen planus group, the leukoplakia group and the normal oral epithelium group. The aforementioned groups were coded as follows:

- A: lichen planus group
- B: leukoplakia group
- D: normal oral epithelium group

The lichen planus group (A) was further divided into the reticular lichen planus subgroup (A1) and the erosive lichen planus subgroup (A2). The leukoplakia group (B) was further divided into the moderately and severely dysplastic leukoplakia subgroup (B1) and the mildly dysplastic and non-dysplastic leukoplakia subgroup (B2) according to the WHO’s 2005 binary classification system for oral leukoplakia [35]. The aforementioned subgroups were coded as follows:

- A1: reticular lichen planus subgroup
- A2: erosive lichen planus subgroup
- B1: moderately and severely dysplastic leukoplakia subgroup
- B2: mildly dysplastic and non-dysplastic leukoplakia subgroup

Each sample was also coded in this manner. Therefore, the samples were coded as follows (Table 1):

- 10 samples of the reticular lichen planus subgroup (A1) that were coded as A1.1–A1.10
- 14 samples of the erosive lichen planus subgroup (A2) that were coded as A2.1–A2.14
- 16 samples of the moderately and severely dysplastic leukoplakia subgroup (B1) that were coded as B1.1–B1.16
- 14 samples of the mildly dysplastic and non-dysplastic leukoplakia subgroup (B2) that were coded as B2.1–B2.14
- 5 samples of the normal oral epithelium group that were coded as D.1–D.5

### 2.2. Immunohistochemistry

The immunohistochemical method required the CSC protein biomarker anti-CD147 (sc-21746, Santa Cruz Biotechnology, Dallas, TX, USA) as well as the Dako Envision Flex+ system (Dako Denmark A/S, Glostrup, Denmark). 

The instruments used for the experiment include the microtome Jung Biocut 2035, the microslides Polysine Superfrost Plus (Thermo scientific Menzel Gläser, Braunschweig, Germany), a microwave oven (Rohnson R-2011, Rohnson, China), the fixed specification binocular biological microscope OLYMPUS CX31 (Olympus LS, Tokyo, Japan) and the 3.3-megapixel-utilizing CMOS chip digital camera Olympus SC30 (Olympus Soft Imaging Solutions, Muenster, Germany). 

More analytically, the quantitative evaluation of immunostaining for the selected antibodies was as follows: The evaluation of the membranous staining of CD147 was obtained as a histochemical score by calculating the percentage of positive cells and then classifying this percentage into a scale from 0 to 3 (Table 2). The staining is deemed to be successful when the cytoplasm or membrane is colored brown.

### 2.3. Statistical Analysis

Statistical analysis was performed using SPSS software (2017) with a Pearson’s chi-square test or Fisher’s exact test depending on the sample size. The significance level was set at 0.05 (*p* = 0.05). 

## 3. Results

Regarding the staining of CD147, all of the samples of the reticular lichen planus subgroup (A1) were scored as 1. One sample of the erosive lichen planus subgroup (A2) was scored as 0, six samples of the erosive lichen planus group (A2) were scored as 1 and seven samples of the erosive lichen planus subgroup (A2) were scored as 2. Seven samples of the moderately and severely dysplastic leukoplakia subgroup (B1) were scored as 0, eight samples of the moderately and severely dysplastic leukoplakia subgroup (B1) were scored as 1 and one sample of the moderately and severely dysplastic leukoplakia subgroup (B1) was scored as 2. All the samples of the mildly dysplastic and non-dysplastic leukoplakia subgroup (B2) were scored as 1. Two samples of the normal oral epithelium group (D) were scored as 0 and three samples of the normal oral epithelium group (D) were scored as 1.

**Figure 1:**
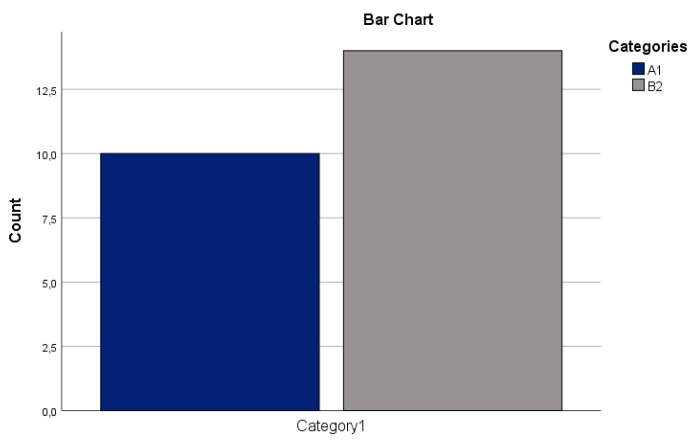
Difference between A1 and B2 regarding CD147. On the y-axis, the number of the samples, stained for CD147, per category is depicted. The categories are A1 (reticular lichen planus, dark blue) and B2 (mildly dysplastic and non-dysplastic leukoplakia, gray). On the x-axis, category 1 represents the score of the samples (all of them were scored as 1).

Therefore, no statistical test was performed between reticular lichen planus and mildly dysplastic and non-dysplastic leukoplakia, since all of their samples were scored as 1 (Figure 1).

**Figure 2:**
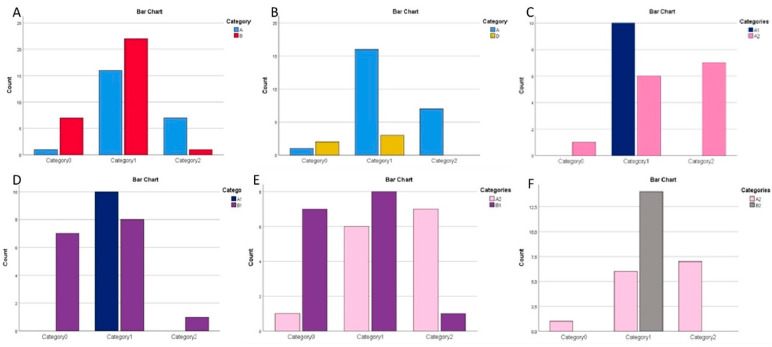
Differences among A, B, D, A1, A2, B1 and B2 regarding CD147. On the y-axis, the number of samples, stained for CD147, per category is depicted. The upper right statistical categories correspond to A (lichen planus, light blue), B (leukoplakia, red), D (normal oral epithelium, yellow), A1 (reticular lichen planus, dark blue), A2 (erosive lichen planus, pink), B1 (moderately and severely dysplastic leukoplakia, mauve) and B2 (mildly dysplastic and non-dysplastic leukoplakia, gray). On the x-axis, category 0 represents the samples scored with 0, category 1 represents the samples scored with 1 and category 2 represents the samples scored with 2. (**A**): Comparison between lichen planus and leukoplakia. (**B**): The comparison between lichen planus and normal oral epithelium. (**C**): Comparison between reticular lichen planus and erosive lichen planus. (**D**): Comparison between reticular lichen planus and moderately and severely dysplastic leukoplakia. (**E**): Comparison between erosive lichen planus and moderately and severely dysplastic leukoplakia. (**F**): Comparison between erosive lichen planus and mildly dysplastic and non-dysplastic leukoplakia.

Based on the histochemical scores and statistical analyses, the following observations emerged (Figure 2):

- Statistically significantly higher expression of CD147 in the lichen planus group than in the leukoplakia group (Pearson’s chi-square, *p* = 0.009) (Figure 2A).
- Statistically significantly higher expression of CD147 in the lichen planus group than in the normal oral epithelium group (Pearson’s chi-square, *p* = 0.036) (Figure 2B).
- Statistically significantly higher expression of CD147 in the erosive lichen planus subgroup than in the reticular lichen planus subgroup (Fisher’s exact test, *p* = 0.006) (Figure 2C).
- Statistically significantly higher expression of CD147 in the reticular lichen planus subgroup than in the moderately and severely dysplastic leukoplakia subgroup (Fisher’s exact test, *p* = 0.014) (Figure 2D).
- Statistically significantly higher expression of CD147 in the erosive lichen planus subgroup than in the moderately and severely dysplastic leukoplakia subgroup (Pearson’s chi-square test, *p* = 0.01) (Figure 2E).
- Statistically significantly higher expression of CD147 in the erosive lichen planus subgroup than in the mildly and non-dysplastic leukoplakia subgroup (Fisher’s exact test, *p* = 0.002) (Figure 2F).

**Figure 3:**
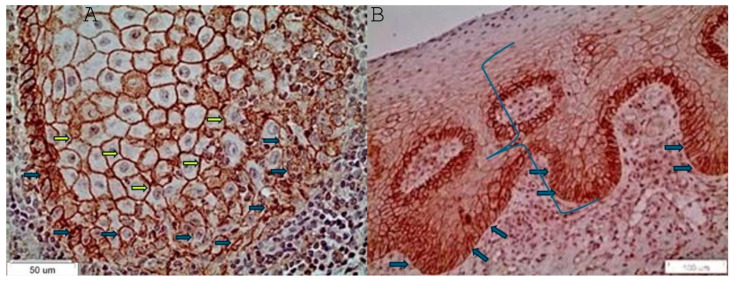
(**A**): Membranous staining of CD147 in the lower third of the epithelium (×40) in a case of reticular lichen planus. The blue arrows show the membranous staining of the basal cell layer. The yellow arrows show the membranous staining of the parabasal cell layer. (**B**): Membranous staining of CD147 in the lower and middle third of the epithelium (×20) in a case of erosive lichen planus. The blue arrows show the membranous staining of the basal cell layer. The blue bracket outlines the positively stained lower and middle third of the epithelium.

The microscopic images below of the reticular lichen planus group and the erosive lichen planus group and their respective descriptions correspond to Figure 3.

## 4. Discussion

The aim of this experimental study was to investigate whether lichen planus expresses CD147 and to what extent compared to leukoplakia and normal epithelium. This goal was addressed by comparing the extent of the presence of CSCs in lichen planus samples to that of cancer stem cells in samples with leukoplakia, a well-established OPMD, and to normal oral epithelium samples. Cancer stem cells mediate the transition of potentially malignant lesions to oral squamous cell carcinoma; therefore, their presence may support the hypothesis that a disorder is potentially malignant. 

The normal epithelium group functioned as the first control group to illustrate the differences between OLP/OLR and the normal epithelium, whereas comparison to OL, a well-established OPMD, is necessary to investigate the potentially malignant nature of OLP and OLR; therefore, the group OL functions as a second control group. CD147 staining could confirm the stemness: the presence of molecular programs that underlie the stem cell state. 

So far, differential diagnosis between OLP and OLR has proven to be unclear. On a histological level, the two entities are indistinguishable. The location of the lesions, the proximity to dental restoration and the symmetrical, intraoral distribution of the lesions were taken into account for the purpose of differential diagnosis. However, these criteria acted as a logical leap since they cannot be characterized as pathognomonic and only functioned on a probabilistic level. Since, by definition, differentiation between OLP and OLR remains unclear, the classification of erosive lichenoid lesions into one group is based solely on their clinical features to avoid confusion regarding their histological features and their histological distinction, and this group may hence be termed as an erosive lichen planus group. 

Immunohistochemistry remains a powerful tool in diagnostic pathology. Successful staining necessitates proper tissue preparation. The traditional fixation method requires fixing samples in formaldehyde and embedding them in paraffin wax prior to microscopic examination. The main advantage of formalin-fixed, paraffin-embedded tissues, and the reason why this fixation method was also employed in our study protocol, is that these well-preserved human tissues may be used in retrospective studies [36,37]. Therefore, all tissue samples received in the past may be utilized. The main disadvantage of this method is the denaturation of epitopes, which leads to the obstruction of the immunohistochemical detection. Therefore, an antigen retrieval method is required. 

A different approach suggests the alternative of snap-frozen tissue sections. The main advantage of snap-frozen tissue sections is that antigen retrieval is usually not required for immunostaining of fresh frozen sections. However, this alternative was not chosen for our study protocol due to its main disadvantage that it must be decided in advance which tissues are frozen for future studies, and there was a complete lack of such tissue samples [37].

The heating, dehydration during paraffin embedding and cross-linking with formaldehyde alter the three-dimensional structure of proteins in paraffin-embedded tissues, leading to the denaturation of antigenic epitopes. Therefore, recovering the antigenicity of fixed tissues constitutes a main research goal.

Initially, antigen retrieval was performed by proteolytic enzymes, which break the formaldehyde-induced methylene cross-links in the antigenic molecules [38]. Proteolytic methods are relatively unpredictable and present the risk of damaging the tissues involved. 

The wet heat-induced epitope retrieval (HIER) method proposes that paraffin-embedded tissues be heated in dilute metal–salt or buffer solutions at or above 100 °C for a duration of several minutes to half an hour and has proved to be of crucial importance in paraffin section immunohistochemistry [39,40,41]. Variations in the original HIER technique are based on differences regarding the buffer solution and the heating mode, preserving the main formula for wet heat treatment over a fixed period of time. The HIER method may be influenced by the composition of the retrieval buffer, the specific heating device used and the temperature and duration of the heat treatment [37]. Based on the aforementioned conclusions, the HIER method was used in our experimental protocol.

Regarding the evaluation of the histochemical score, CD147 was measured only on a quantitative level by calculating the percentage of positive cells because the same intensity of staining was observed in all our samples and a qualitative evaluation was deemed to be unnecessary. 

Additionally, the presence of controls in each batch of tissue sections submitted to the immunohistochemical technique was required so as to act as an internal control. 

Finally, the Dako Envision Flex and immunohistochemistry kit created clear immunohistochemical staining, which allowed photographs to be taken under an optical microscope.

During the 19th century, it was observed that sites of chronic inflammation gave rise to tumors and that inflammatory cells were present in tumor tissues [42]. Therefore, the presence of chronic inflammation was associated with carcinogenesis. The interconnection among tumor cells, non-malignant stromal cells and the extracellular matrix of the tumor microenvironment enables the tumor to primarily invade in situ and subsequently metastasize [43]. The extracellular matrix is reorganized and broken down during inflammation [44] and this breakdown is mediated by proteolytic enzymes, of which the most crucial, in terms of carcinogenesis, is the matrix metalloproteinase (MMP) group of enzymes [45]. MMPs concentrate on the invadopodia, thus enabling cancer invasion into adjacent stroma and facilitating distant metastasis [33]. Generally, CD147 stimulates the formation of MMPs and participates in invasion [46]. CD147 activates MMP-1 and MMP-9, possibly leading to intrabony invasion [46]. Another study showed that MMP and CD147 are associated with bone invasion [47]. CD147 activation of MMPs in the tumor microenvironment enables tumor cells undergoing EMT to invade the surrounding stroma [48]. 

The results of our study include the following observations:

Moderately and severely dysplastic leukoplakia is a well-known potentially malignant oral disorder, which expressed the biomarker CD147 less than the erosive lichen planus group. This indicates that inflammation mediated by CD147 is present in erosive oral lichen planus and may trigger its malignant transformation. A similar study of our research collaboration investigated the expression of ALDH1 and 2 in OLP compared to OL [49]. In this previous study, the moderately and severely dysplastic leukoplakia expressed the biomarker ALDH1 and 2 similarly to the erosive lichen planus. Since OLP is distinguished by the production of cytokines associated with inflammation, these cytokines may initiate a continuous inflammatory response [50]. Hence, it is possible that the manifestation of CD147 and ALDH signifies the existence of both CSCs and underlying inflammation. The coexistence of ALDH1 and 2 with CD147 in the same tissue samples of erosive lichen planus and moderately and severely dysplastic leukoplakia indicates the presence of triangular intracorrelation among CSC biomarkers’ expression, inflammation and the malignant transformation of OPMDs. The mildly and non-dysplastic leukoplakia group expressed CD147 similarly to the reticular lichen planus group (same findings regarding ALDH1 and 2 in our previous study). Furthermore, the erosive lichen planus group expressed more CD147 than the reticular lichen planus group, suggesting the presence of a more intense inflammatory microenvironment in erosive OLP (contradicting our previous findings regarding erosive and reticular OLP, where both entities expressed ALDH1 and 2 to the same extent [49]). Further studies are required to illustrate whether inflammation constitutes the main instigator of malignant transformation in OLP or if the malignant potential is preexisting and inflammation plays a role in refueling this malignant potential. The limitations of this study include a relatively small sample size due to a lack of adequate paraffin-embedded tissues.

## 5. Conclusions

CD147 may be more sensitive to inflammation than dysplasia. CD147 expression influences the transition to a premalignant state of oral potentially malignant disorders through associated inflammation. The characteristic expression of CD147 in erosive OLP suggests the presence of epithelial cells with CSC characteristics, whereas its lower expression in oral leukoplakia suggests a more intense relation of the CD147 marker to inflammation, rather than oral dysplastic progression. This is the first original research article in the literature investigating the presence of CD147 in oral potentially malignant disorders.

## Figures and Tables

**Table 1:** Overview of the samples, including the grouping, coding, location of the lesions and the age and gender of the samples’ respective patients.

Patients	Group/Subgroup	Location	Gender	Age
A1.1	RETICULAR OLP	TONGUE	MALE	57
A1.2	RETICULAR OLP	TONGUE	MALE	77
A1.3	RETICULAR OLP	TONGUE	FEMALE	21
A1.4	RETICULAR OLP	TONGUE	FEMALE	50
A1.5	RETICULAR OLP	TONGUE	FEMALE	57
A1.6	RETICULAR OLP	BUCCAL MUCOSA	FEMALE	72
A1.7	RETICULAR OLP	TONGUE	FEMALE	38
A1.8	RETICULAR OLP	BUCCAL MUCOSA	MALE	73
A1.9	RETICULAR OLP	BUCCAL MUCOSA	FEMALE	49
A1.10	RETICULAR OLP	CHEEK	FEMALE	42
A2.1	EROSIVE OLP	INTERDENTAL PAPILLA	FEMALE	77
A2.2	EROSIVE OLP	BUCCAL MUCOSA	FEMALE	77
A2.3	EROSIVE OLP	TONGUE	FEMALE	59
A2.4	EROSIVE OLP	BUCCAL MUCOSA	FEMALE	54
A2.5	EROSIVE OLP	PALATE	FEMALE	55
A2.6	EROSIVE OLP	BUCCAL MUCOSA	FEMALE	49
A2.7	EROSIVE OLP	BUCCAL MUCOSA	FEMALE	72
A2.8	EROSIVE OLP	BUCCAL MUCOSA	FEMALE	76
A2.9	EROSIVE OLP	BUCCAL MUCOSA	FEMALE	58
A2.10	EROSIVE OLP	BUCCAL MUCOSA	FEMALE	64
A2.11	EROSIVE OLP	BUCCAL MUCOSA	MALE	56
A2.12	EROSIVE OLP	BUCCAL MUCOSA	MALE	56
A2.13	EROSIVE OLP	GINGIVA	FEMALE	26
A2.14	EROSIVE OLP	BUCCAL MUCOSA	FEMALE	37
B1.1	MODERATELY AND SEVERELY DYSPLASTIC OL	TONGUE	FEMALE	44
B1.2	MODERATELY AND SEVERELY DYSPLASTIC OL	TONGUE	FEMALE	60
B1.3	MODERATELY AND SEVERELY DYSPLASTIC OL	TONGUE	MALE	58
B1.4	MODERATELY AND SEVERELY DYSPLASTIC OL	TONGUE	FEMALE	67
B1.5	MODERATELY AND SEVERELY DYSPLASTIC OL	TONGUE	FEMALE	62
B1.6	MODERATELY AND SEVERELY DYSPLASTIC OL	BUCCAL MUCOSA	MALE	66
B1.7	MODERATELY AND SEVERELY DYSPLASTIC OL	BUCCAL MUCOSA	MALE	67
B1.8	MODERATELY AND SEVERELY DYSPLASTIC OL	TONGUE	MALE	43
B1.9	MODERATELY AND SEVERELY DYSPLASTIC OL	GINGIVOBUCCAL SULCUS	FEMALE	75
B1.10	MODERATELY AND SEVERELY DYSPLASTIC OL	TONGUE	MALE	50
B1.11	MODERATELY AND SEVERELY DYSPLASTIC OL	GINGIVOBUCCAL SULCUS	MALE	59
B1.12	MODERATELY AND SEVERELY DYSPLASTIC OL	TONGUE	MALE	75
B1.13	MODERATELY AND SEVERELY DYSPLASTIC OL	TONGUE	MALE	64
B1.14	MODERATELY AND SEVERELY DYSPLASTIC OL	TONGUE	MALE	45
B1.15	MODERATELY AND SEVERELY DYSPLASTIC OL	PALATE	MALE	72
B1.16	MODERATELY AND SEVERELY DYSPLASTIC OL	TONGUE	FEMALE	84
B2.1	MILDLY DYSPLASTIC AND NON-DYSPLASTIC OL	TONGUE	FEMALE	61
B2.1	MILDLY DYSPLASTIC AND NON-DYSPLASTIC OL	LIP	FEMALE	38
B2.3	MILDLY DYSPLASTIC AND NON-DYSPLASTIC OL	TONGUE	MALE	46
B2.4	MILDLY DYSPLASTIC AND NON-DYSPLASTIC OL	GINGIVA	FEMALE	12
B2.5	MILDLY DYSPLASTIC AND NON-DYSPLASTIC OL	TONGUE	FEMALE	45
B2.6	MILDLY DYSPLASTIC AND NON-DYSPLASTIC OL	TONGUE	MALE	67
B2.7	MILDLY DYSPLASTIC AND NON-DYSPLASTIC OL	BUCCAL MUCOSA	FEMALE	60
B2.8	MILDLY DYSPLASTIC AND NON-DYSPLASTIC OL	TONGUE	FEMALE	68
B2.9	MILDLY DYSPLASTIC AND NON-DYSPLASTIC OL	TONGUE	MALE	69
B2.10	MILDLY DYSPLASTIC AND NON-DYSPLASTIC OL	TONGUE	FEMALE	68
B2.11	MILDLY DYSPLASTIC AND NON-DYSPLASTIC OL	BUCCAL MUCOSA	FEMALE	58
B2.12	MILDLY DYSPLASTIC AND NON-DYSPLASTIC OL	BUCCAL MUCOSA	FEMALE	61
B2.13	MILDLY DYSPLASTIC AND NON-DYSPLASTIC OL	BUCCAL MUCOSA	MALE	75
B2.14	MILDLY DYSPLASTIC AND NON-DYSPLASTIC OL	CORNER OF THE MOUTH	MALE	37
D.1	NORMAL	TONGUE	FEMALE	49
D.2	NORMAL	BUCCAL MUCOSA	MALE	81
D.3	NORMAL	BUCCAL MUCOSA	FEMALE	59
D.4	NORMAL	TONGUE	MALE	69
D.5	NORMAL	TONGUE	FEMALE	72

**Table 2:** Histochemical score of CD147.

0–5%	0
6–35%	1
36–70%	2
>71%	3

## Data Availability

The original data presented in the study are included in the article, further inquiries can be directed to the corresponding author.

## References

[1] van der Waal I. (2009). Potentially malignant disorders of the oral and oropharyngeal mucosa; terminology, classification and present concepts of management. Oral Oncol..

[2] van der Waal I. (2014). Oral potentially malignant disorders: Is malignant transformation predictable and preventable?. Med. Oral Patol. Oral Y Cirugía Bucal.

[3] Schmidt-Westhausen A.M. (2020). Oral lichen planus and lichenoid lesions: What’s new?. Quintessence Int..

[4] Jaafari-Ashkavandi Z., Mardani M., Pardis S., Amanpour S. (2011). Oral mucocutaneous diseases: Clinicopathologic analysis and malignant transformation. J. Craniofacial Surg..

[5] Rimkevičius A., Aleksejūnienė J., Pūrienė A., Šeinin D., Rastenienė R. (2017). Oral lichen planus: A 4-year clinical follow-up study. Turk. J. Med. Sci..

[6] Dudhia B., Dudhia S., Patel P., Jani Y. (2015). Oral lichen planus to oral lichenoid lesions: Evolution or revolution. J. Oral Maxillofac. Pathol..

[7] Laeijendecker R., van Joost T., Tank B., Oranje A.P., Neumann H.A.M. (2005). Oral lichen planus in childhood. Pediatr. Dermatol..

[8] Patel S., Yeoman C.M., Murphy R. (2005). Oral lichen planus in childhood: A report of three cases. Int. J. Paediatr. Dent..

[9] Gümrü B. (2013). A retrospective study of 370 patients with oral lichen planus in Turkey. Med. Oral Patol. Oral Y Cirugía Bucal.

[10] Gupta S., Jawanda M.K. (2015). Oral lichen planus: An update on etiology, pathogenesis, clinical presentation, diagnosis and management. Indian J. Dermatol..

[11] de Sousa F.A.C.G., Rosa L.E.B. (2008). Oral lichen planus: Clinical and histopathological considerations. Rev. Bras. De Otorrinolaringol..

[12] Gonzalez-Moles M.A., Scully C., Gil-Montoya J.A. (2008). Oral lichen planus: Controversies surrounding malignant transformation. Oral Dis..

[13] Mattsson U., Jontell M., Holmstrup P. (2002). Oral lichen planus and malignant transformation: Is a recall of patients justified?. Crit. Rev. Oral Biol. Med..

[14] Valente G., Pagano M., Carrozzo M., Carbone M., Bobba V., Palestro G., Gandolfo S. (2001). Sequential immunohistochemical p53 expression in biopsies of oral lichen planus undergoing malignant evolution. J. Oral Pathol. Med..

[15] Muzio L.L., Mignogna M.D., Favia G., Procaccini M., Testa N.F., Bucci E. (1998). The possible association between oral lichen planus and oral squamous cell carcinoma: A clinical evaluation on 14 cases and a review of the literature. Oral Oncol..

[16] van der Meij E.H., Schepman K.P., Smeele L.E., van der Wal J.E., Bezemer P.D., van der Waal I. (1999). A review of the recent literature regarding malignant transformation of oral lichen planus. Oral Surg. Oral Med. Oral Pathol. Oral Radiol. Endodontol..

[17] Mignogna M.D., Muzio L.L., Russo L.L., Fedele S., Ruoppo E., Bucci E. (2001). Clinical guidelines in early detection of oral squamous cell carcinoma arising in oral lichen planus: A 5-year experience. Oral Oncol..

[18] Mignogna M.D., Russo L.L., Fedele S., Ruoppo E., Califano L., Muzio L.L. (2002). Clinical behaviour of malignant transforming oral lichen planus. Eur. J. Surg. Oncol..

[19] Abbate G., Foscolo A.M., Gallotti M., Lancella A., Mingo F. (2006). Neoplastic transformation of oral lichen: Case report and review of the literature. Acta Otorhinolaryngol. Ital..

[20] Georgakopoulou E.A., Achtari M.D., Achtaris M., Foukas P.G., Kotsinas A. (2012). Oral lichen planus as a preneoplastic inflammatory model. BioMed Res. Int..

[21] Scully C., Beyli M., Ferreiro M.C., Ficarra G., Gill Y., Griffiths M., Holmstrup P., Mutlu S., Porter S., Wray D. (1998). Update on oral lichen planus: Etiopathogenesis and management. Crit. Rev. Oral Biol. Med..

[22] Albrecht M., Bánóczy J., Dinya E., Tamás G. (1992). Occurrence of oral leukoplakia and lichen planus in diabetes mellitus. J. Oral Pathol. Med..

[23] Lundström I.M.C. (1983). Incidence of diabetes mellitus in patients with oral lichen planus. Int. J. Oral Surg..

[24] Torrente-Castells E., Figueiredo R., Berini-Aytés L., Gay-Escoda C. (2010). Clinical features of oral lichen planus. A retrospective study of 65 cases. Med. Oral Patol. Oral Y Cir. Bucal.

[25] Lamey P.J., Gibson J., Barclay S.C., Miller S. (1990). Grinspan’s syndrome: A drug-induced phenomenon?. Oral Surg. Oral Med. Oral Pathol..

[26] Sharma N., Malhotra S.K., Kuthial M., Chahal K.S. (2017). Vulvo-vaginal ano-gingival syndrome: Another variant of mucosal lichen planus. Indian J. Sex. Transm. Dis. AIDS.

[27] Birkenfeld S., Dreiher J., Weitzman D., Cohen A.D. (2011). A study on the association with hepatitis B and hepatitis C in 1557 patients with lichen planus. J. Eur. Acad. Dermatol. Venereol..

[28] Campisi G., Giovannelli L., Aricò P., Lama A., Di Liberto C., Ammatuna P., D’Angelo M. (2004). HPV DNA in clinically different variants of oral leukoplakia and lichen planus. Oral Surg. Oral Med. Oral Pathol. Oral Radiol. Endodontol..

[29] Gorsky M., Epstein J.B. (2011). Oral lichen planus: Malignant transformation and human papilloma virus: A review of potential clinical implications. Oral Surg. Oral Med. Oral Pathol. Oral Radiol. Endodontol..

[30] Kumari R., Singh N., Thappa D.M. (2009). Hypertrophic lichen planus as a presenting feature of human immunodeficiency virus infection. Indian J. Dermatol..

[31] Sand L.P., Jalouli J., Larsson P.A., Hirsch J.M. (2002). Prevalence of Epstein-Barr virus in oral squamous cell carcinoma, oral lichen planus, and normal oral mucosa. Oral Surg. Oral Med. Oral Pathol. Oral Radiol. Endodontol..

[32] Yildirim B., Sengüven B., Demir C. (2011). Prevalence of herpes simplex, Epstein Barr and human Papilloma viruses in oral lichen planus. Med. Oral Patol Oral Cir Bucal.

[33] Nasry W.H.S., Rodriguez-Lecompte J.C., Martin C.K. (2018). Role of COX-2/PGE2 mediated inflammation in oral squamous cell carcinoma. Cancers.

[34] Monteiro L.S., Delgado M.L., Ricardo S., Garcez F., Amaral B.D., Pacheco J.J., Bousbaa H. (2014). EMMPRIN expression in oral squamous cell carcinomas: Correlation with tumor proliferation and patient survival. BioMed Res. Int..

[35] Warnakulasuriya S., Reibel J., Bouquot J., Dabelsteen E. (2008). Oral epithelial dysplasia classification systems: Predictive value, utility, weaknesses and scope for improvement. J. Oral Pathol. Med..

[36] Mason D.Y., Gatter K.C. (1987). The role of immunocytochemistry in diagnostic pathology. J. Clin. Pathol..

[37] Krenacs L., Krenacs T., Stelkovics E., Raffeld M. (2010). Heat-Induced Antigen Retrieval for Immunohistochemical Reactions in Routinely Processed Paraffin Sections. Immunocytochemical Methods Protoc..

[38] Battifora H., Kopinski M. (1986). The influence of protease digestion and duration of fixation on the immunostaining of keratins. A comparison of formalin and ethanol fixation. J. Histochem. Cytochem..

[39] Cattoretti G., Pileri S., Parravicini C., Becker M.H., Poggi S., Bifulco C., Rilke F. (1993). Antigen unmasking on formalin-fixed, paraffin-embedded tissue sections. J. Pathol..

[40] Norton A.J., Jordan S., Yeomans P. (1994). Brief, high-temperature heat denaturation (pressure cooking): A simple and effective method of antigen retrieval for routinely processed tissues. J. Pathol..

[41] Shi S.R., Key M.E., Kalra K.L. (1991). Antigen retrieval in formalin-fixed, paraffin-embedded tissues: An enhancement method for immunohistochemical staining based on microwave oven heating of tissue sections. J. Histochem. Cytochem..

[42] Sarode S.C., Sarode G.S., Kalele K. (2012). Oral Lichenoid Reaction: A Review. Int. J. Oral Maxillofac. Pathol..

[43] Coghlin C., Murray G.I. (2014). The role of gene regulatory networks in promoting cancer progression and metastasis. Future Oncol..

[44] Bonnans C., Chou J., Werb Z. (2014). Remodelling the extracellular matrix in development and disease. Nat. Rev. Mol. Cell Biol..

[45] Curran S., Murray G.I. (2000). Matrix metalloproteinasesmolecular aspects of their roles in tumour invasion and metastasis. Eur. J. Cancer.

[46] Erdem N.F., Carlson E.R., Gerard D.A., Ichiki A.T. (2007). Characterization of 3 Oral Squamous Cell Carcinoma Cell Lines With Different Invasion and/or Metastatic Potentials. J. Oral Maxillofac. Surg..

[47] De Vicente J.C., Fresno M.F., Villalain L., Vega J.A., Vallejo G.H. (2005). Expression and clinical significance of matrix metalloproteinase-2 and matrix metalloproteinase-9 in oral squamous cell carcinoma. Oral Oncol..

[48] Papadimitropoulou A., Mamalaki A. (2013). The glycosylated IgII Extracellular domain of EMMPRIN is implicated in the induction of MMP-2. Mol. Cell. Biochem..

[49] Zisis V., Giannakopoulos N.N., Schmitter M., Poulopoulos A., Andreadis D. (2023). Cancer Stem Cells’ Biomarker ALDH1&2 Increased Expression in Erosive Oral Lichen Planus Compared to Oral Leukoplakia. Cureus.

[50] Lu R., Zhang J., Sun W., Du G., Zhou G. (2015). Inflammation-related cytokines in oral lichen planus: An overview. J. Oral Pathol. Med..

